# Unlocking the potential of neuromuscular electrical stimulation: achieving physical activity benefits for all abilities

**DOI:** 10.3389/fspor.2024.1507402

**Published:** 2024-11-29

**Authors:** Paul W. Ackermann, Robin Juthberg, Johanna Flodin

**Affiliations:** ^1^ Integrative Orthopedic Laboratory, Department of Molecular Medicine and Surgery, Karolinska Institutet, Stockholm, Sweden; ^2^ Department of Trauma, Acute Surgery and Orthopedics, Karolinska University Hospital, Stockholm, Sweden

**Keywords:** neuromuscular electrical stimulation, immobilization, muscle strengthening, exercise, blood flow, venous thromboembolism, motor points

## Abstract

Neuromuscular Electrical Stimulation (NMES) uses electrical impulses to induce muscle contractions, providing benefits in rehabilitation, muscle activation, and as an adjunct to exercise, particularly for individuals experiencing immobilization or physical disability. NMES technology has significantly progressed, with advancements in device development and a deeper understanding of treatment parameters, such as frequency, intensity, and pulse duration. These improvements have expanded NMES applications beyond rehabilitation to include enhanced post-exercise recovery, improved blood glucose uptake, and increased lower limb venous return, potentially reducing thrombotic risks. Despite its benefits, NMES faces challenges in user compliance, often due to improper electrode placement and discomfort during treatment. Research highlights the importance of optimizing stimulation parameters, including electrode positioning, to improve both comfort and treatment efficacy. Recent innovations, such as automated processes for locating optimal stimulation points and adaptable electrode sizes, aim to address these issues. When combined with wearable technologies, these innovations could improve NMES treatment adherence and deliver more consistent, long-term therapeutic outcomes for patients with various physical limitations. Together, these developments indicate a promising future for NMES, presenting a valuable tool to enhance the benefits of physical activity across diverse populations, from rehabilitative care to broader health and wellness applications.

## Introduction

Neuromuscular electrical stimulation (NMES) is a treatment method used to create muscle contractions through electrical impulses. NMES mimics the body's nervous system during voluntary muscle activation, but instead of the signal originating in the brain, it comes from an electrical stimulator. This is achieved by placing electrodes on the skin over the target muscle.

The use of electricity for medical treatment dates back to ancient Egypt and Greece, where electric eels were used for pain relief. Modern NMES evolves from Galvani’s 18th century discovery that electric current can induce muscle contraction (1). Today, electrical stimulation is applied in various medical contexts, such as transcutaneous electrical nerve stimulation (TENS) for pain management (2) and NMES in rehabilitation settings. NMES is commonly used to strengthen weakened muscles, reduce muscle atrophy during immobilization (e.g., after surgery or injury), and complement exercise to optimize training effects (3, 4).

However, current NMES protocols still suffer from poor compliance and inadequate efficacy, attributed to limited/insufficient user proficiency regarding repeated application of electrodes in the correct placement. Research has shown that electrode placement based on a prior manual search of the optimal points and individual adaption of electrode dimensions significantly improves treatment effectiveness and comfort (5).

Recent innovations have introduced an automated search process that identifies the optimal stimulation points and electrode sizes for each patient, ensuring consistent results and improved compliance with NMES treatment (6). Combined with wearable technologies, such as garment-based applications, these advancements hold the potential to enhance treatment adherence and improve long-term outcomes for patients with physical disabilities.

### General considerations of NMES usage

#### Settings for optimal NMES usage

**Figure 1:**
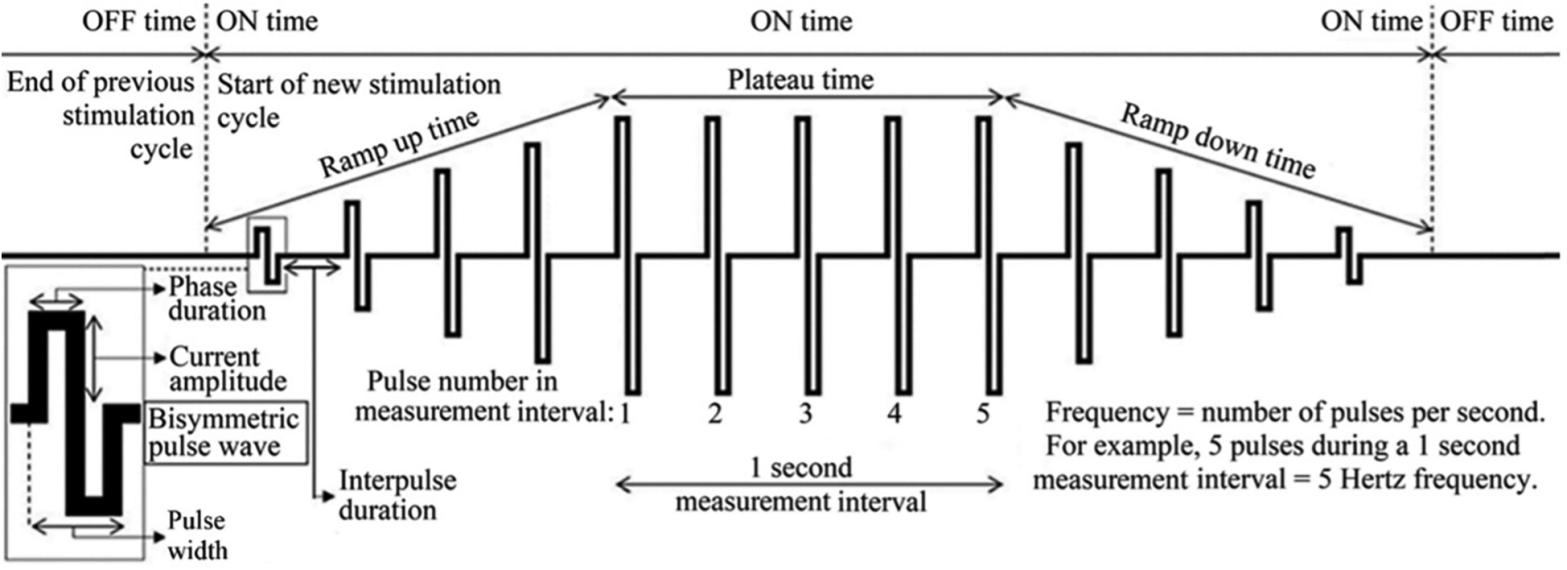
Examples of parameters that may be adjusted for optimal treatment effects and comfort during neuromuscular electrical stimulation (NMES). Image adapted from Juthberg et al. (10).

Studies have shown that the NMES parameters also affect the comfort and effectiveness of the stimulation (3, 7–9). Several parameters can be adjusted during NMES, such as frequency, pulse width, intensity, waveform, plateau time, on:off-time and ramp-up/ramp-down time (Figure 1).

##### Pulse width, frequency, and intensity

Overall, research has shown that increasing each of pulse width, frequency, and/or intensity leads to recruitment of more muscle fibers, resulting in higher force production (7–10). However, these parameters also affect the comfort of stimulation. Frequency correlates directly with muscle torque production, even when using textile electrodes (12). Longer pulse widths, such as between 400 and 600 μs, selectively target motor fibers, while shorter pulse widths target sensory fibers to a higher extent. Consequently, longer pulse widths positively influences muscle torque production. Studies on quadriceps NMES suggest that a pulse width of 400–600 µs and frequency between 30 and 50 Hz provide optimal muscle recruitment without inducing excessive muscle fatigue and metabolic demands (3).

##### On: off-time and ramp-up/ramp-down time

The on: off-time and ramp-up/ramp-down time are believed to mainly affect comfort. The optimal on: off time is not well defined, but if the off-time is too short, the risk of fatigue increases due to insufficient muscle recovery (3). Prior research has demonstrated that an on: off time of 1:5 (e.g., 10 s on and 50 s off) allows the muscle to recover between stimulations (3).

##### High- and low-intensity NMES

Most research has focused on high-intensity NMES, aiming to mimic the muscle contractions during maximal exercise, which is desirable for muscle strengthening effects (13, 14). However, high intensity NMES can be quite painful. In contrast, low-intensity (LI)-NMES results in minimal pain (15) while still producing muscle contractions, an outcome which significantly increases both venous and arterial blood flow. LI-NMES has therefore been demonstrated as a promising method to prevent venous thromboembolism in both calf and quadriceps muscles (12, 16–18).

#### Differences between NMES and voluntary muscle contractions

While NMES aims to mimic voluntary muscle contraction, it differs from the contractions induced via the central nervous system in a number of ways. NMES activates muscle units simultaneously between the position of the electrodes, often targeting superficial muscles, and recruits them repetitively in a fixed spatial pattern, which leads to quicker fatigue compared to voluntary contractions (3, 7–9). In contrast, voluntary muscle contractions disperse the recruitment of motor units and vary their activation in numbers and across changing locations. NMES also primarily targets fast-twitch muscle fibers, which contribute to quicker fatigue but on the other hand is advantageous for rehabilitation, as these are the fibers predominantly weakened following injury or surgery (3).

#### Side effects of NMES

In recent years, an increasing number of studies have indicated that improper electrical stimulation can have harmful effects (19). In addition to common muscle soreness lasting one to four days after treatment, over-treatment can result in muscle fiber damage, increased secretion of creatine kinase, and muscle breakdown (rhabdomyolysis). This can potentially lead to acute kidney failure, especially in individuals whose kidney function already is reduced (20). These injuries have been particularly noted with excessive muscle training in suits, i.e., whole-body electromyostimulation, containing many electrodes stimulating several muscle groups simultaneously (20). However, whole-body electromyostimulation has in recent reviews of controlled trials shown significant, moderate to large effect sizes on sarcopenia, muscle mass and strength parameters (21).

**Figure 2:**
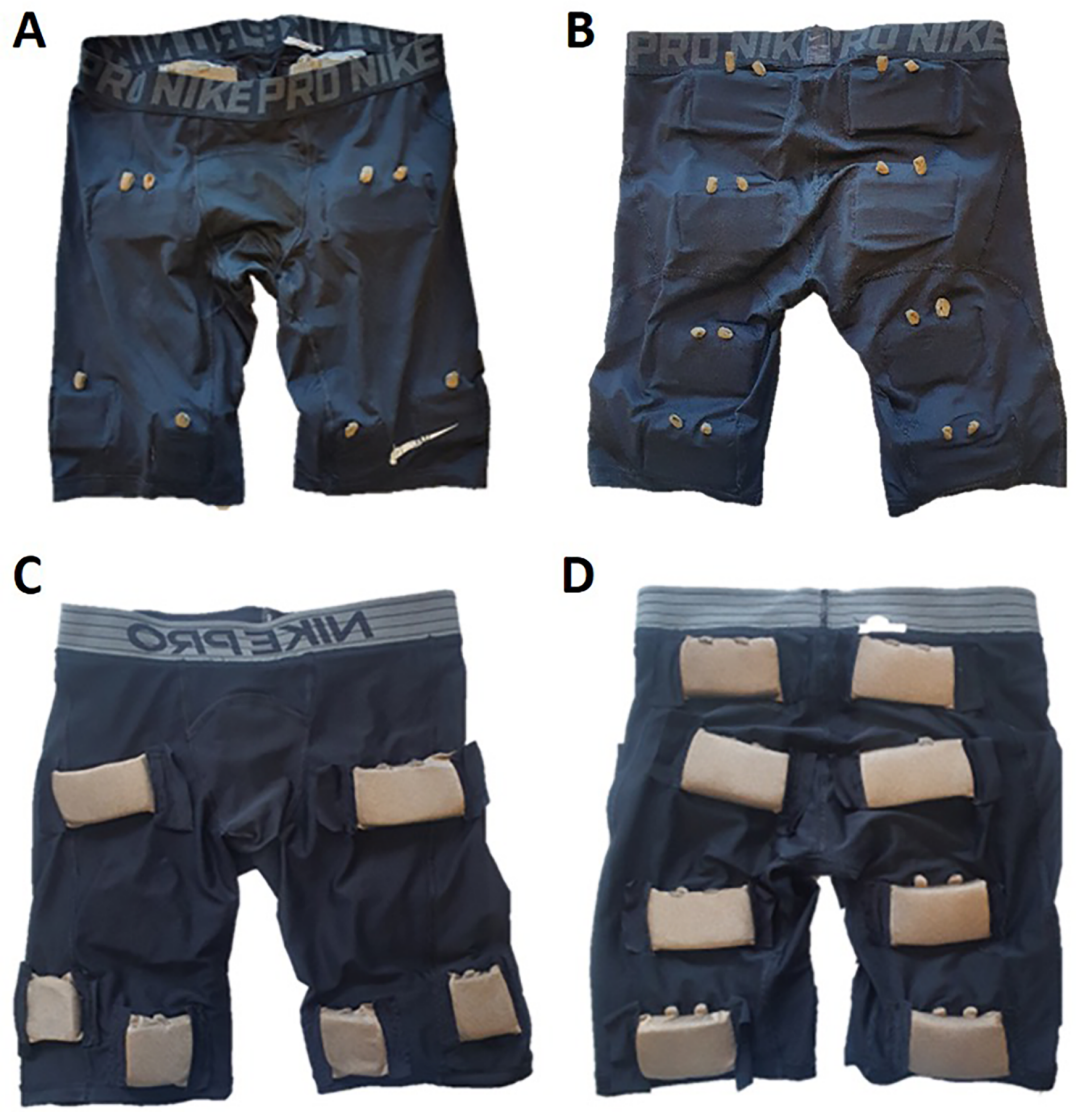
Image of NMES pants seen from the outside (**A** frontside, **B** backside) and inside (**C** frontside, **D** backside). On **(A,B)** there are connectors for stimulation. The larger electrodes are sized 5 × 9 cm (upper electrodes in **C** and all electrodes in **D**) and the smaller (lower electrodes in **C**) are sized 5 × 5 cm. The NMES pants are developed together with the Swedish School of Textiles at the University of Borås, Sweden. Image adapted from (12).

Other side effects or drawbacks of current NMES treatments are that many patients experience discomfort or pain during stimulation (3, 4), and difficulties in correctly setting up the NMES device without assistance, leading to low adherence (22, 23). Adherence to treatment is the most important factor in determining whether the treatment in clinical practice can achieve the effects shown in studies. This challenge has prompted researchers at Karolinska Institutet to focus on the development and optimization of NMES, including integrating the treatment into clothing, which has the potential to dramatically improve treatment adherence with NMES (Figure 2) (24).

#### NMES electrodes

Optimal NMES usage requires adapting the size, number, and placement of electrodes (Figure 2). Studies have shown that larger electrodes are more comfortable than smaller ones, but excessively large electrodes decrease the effectiveness of the treatment (15, 25). The optimal size also depends on the muscle being stimulated, with larger muscles generally requiring larger electrodes (15, 25). Placement of electrodes on so-called motor points has been shown to provide more comfortable and effective stimulation (3, 5). The electrophysiological definition of a motor point is the location on the skin which requires the lowest intensity of electrical stimulation to cause a muscle contraction (26). However, the time-consuming manual motor point search with a motor point pen requires training, and thus poses a problem in the daily use of NMES. Therefore, anatomical maps have been developed showing where on the body motor points are most likely to be found (5, 27, 28). However, there is significant individual variation in the location of motor points and thus normal users will experience problems in locating motor points, which will lead to a compliance problem with the therapy.

##### Multiple NMES electrodes in a matrix

To address these challenges and account for the large individual variation in motor point locations, years of development has led to an automatic motor point search procedure performed within a matrix of electrodes, which may improve NMES treatment adherence (6). Within a matrix of electrodes, multiple electrodes can be combined to form individualized electrode sizes, enhancing both compliance and treatment outcomes. Customizing electrode sizes is essential for different treatment indications and muscle groups. Additionally, varying stimulation within the electrode matrix can reduce muscle fatigue and recruit a broader range of muscle fibers. As a result, NMES within an electrode matrix offers the potential for automatic electrode placement over motor points, with individualized electrode sizes and numbers tailored to specific treatments.

### Muscle strengthening effects of NMES

NMES is currently used both as a complement to training to optimize the effect in healthy individuals and by physiotherapists as part of rehabilitation, such as after surgery or injuries, to maintain muscle strength and strengthen weakened muscles (3, 4, 27–29). Most earlier studies on the training effects of NMES have focused on its use alongside voluntary muscle activation (30, 31). However, recent studies show that NMES alone can also have beneficial effects on maintaining muscle strength, rehabilitating injuries, and achieving recovery after training (3, 32).

#### NMES with voluntary muscle contraction

Several studies have demonstrated that combining NMES with voluntary muscle contractions yields superior training effects, both in younger and older individuals (3, 9, 29–31). For example, one study demonstrated that NMES combined with leg and gluteal strength training three times a week during for four weeks in older adults improved walking test times more than exercise alone (33). Another study in young, healthy and physically active individuals showed that a 6-week training program involving vertical jumps with added electrical stimulation increased jump height by 10% more than just jump training alone or no training at all (3). These findings suggest that NMES can stimulate parts of the muscle that regular exercise may not easily reach and/or provide a greater load the than typical training. Moreover, a recent study indicated that NMES and exercise can potentiate each other even when performed on opposite extremities (34). These observations suggest involvement of either systemic mediator mechanisms and/or effects mediated through the central nervous system.

#### NMES without voluntary muscle contraction

Studies have shown that NMES alone can achieve a relatively high percentage of the maximum force generated during voluntary muscle activation, without any voluntary contraction (3, 9, 35, 36). To measure the force generated during knee extension exercises of the thigh muscles, a dynamometer (Biodex) is used, and the maximum force generated is referred to as the “maximum voluntary contraction” (MVC) (37). The degree of muscle activation during NMES can also be measured using this device (36), and the percentage of MVC achieved is reported as a percentage of MVC.

Research suggests that a stimulation level of at least 20% of MVC is required for muscle-strengthening effects (9). Muscle contractions induced by NMES generally produce lower force output than voluntary contractions, usually less than 50% of MVC at the highest tolerable intensity (38), which is attributed to the difference between how the muscles are activated (3, 8, 9). NMES may also activate more superficial muscle fibers, which could result in poorer training outcomes compared to regular exercise. This has led to further research aimed at improving NMES techniques (38), including optimizing the number, placement, and size of electrodes, NMES parameters, and training protocols (8, 15, 23, 39). Additionally, combining NMES with methods like blood flow restriction has shown potential for producing better training effects than NMES alone (38, 40).

#### The molecular effects of NMES

To better understand the effects of NMES on muscle, several studies have examined its effects at the gene and muscle fiber levels (13, 41–45). One study comparing a 30-min NMES session at the highest tolerable intensity with regular strength training found that both methods altered the expression of genes activated by exercise in the thigh muscle 24 h post-workout (13). While regular strength exercise regulated gene expression to a greater extent than NMES, NMES remains a good alternative when regular exercise is not possible (13).

A recent study conducted by our research group demonstrated that a single NMES session at 20% MVC, using NMES pants, regulated 4,448 differentially expressed genes (DEGs), with an 80% overlap with the 2,571 DEGs regulated by regular exercise. The genes regulated by NMES included well-known exercise-related genes such as PPARGC1A, ABRA, VEGFA, and GDNF. Only eight genes were regulated in opposite directions by NMES and exercise. The three genes upregulated by NMES and downregulated by exercise included genes involved in neurite outgrowth (MYLIP), cell proliferation and regulation of mTORC1 signaling (ICK) and negative regulation of cell proliferation (JARID2) (34). It was also demonstrated that the NMES-session at 20% of MVC could be applied with an acceptable level of discomfort, e.g., VAS below 4 (34).

In other studies, the effect of multiple NMES treatments (over 5 days to 10 weeks, with 3–6 sessions per week, lasting 18 min to 2 h per session) have been shown to affect gene and muscle fiber composition in both younger (41, 43) and older adults (41, 44). These effects have also been observed orthopedic contexts, aiding recovery of quadriceps strength after knee surgery, anterior cruciate ligament reconstruction and total knee arthroplasty (42, 46–48).

In summary, studies have concluded that NMES can preserve muscle mass, prevent muscle atrophy, and to some extent alter and improve gene expression. When compared to regular exercise, the effects of NMES are less pronounced, but it remains a valuable option for individuals unable to engage in regular exercise and as a complement to standard training for healthy individuals (41, 42).

### NMES benefits during physical inactivity

Physical inactivity is a major and growing global health problem, contributing to approximately 3.2 million preventable deaths each year (49, 50). Immobilization and inactivity are closely linked to the development and progression of obesity, type 2 diabetes (50), venous blood clot development in the legs and lungs (51), and reduced muscle strength and balance, which can lead to falls, particularly in older adults (49, 50, 52). While physical activity is an effective way to counteract these negative effects, it is not always possible, especially after surgery or for older patients with underlying illnesses (53). Current treatment methods for these conditions are often insufficient, largely due to low compliance. There is a clear need for improved treatment options to mitigate the adverse effects of physical inactivity. NMES, which uses the body's own energy to create muscle contractions, is an alternative way to activate muscles during periods of immobilization (30, 54–56). This makes NMES especially beneficial for older adults, post-surgical patients, and individuals with cardiovascular risks or other co-morbidities, such as chronic obstructive pulmonary disease, who have difficulty engaging with exercise programs.

#### Improved balance

For people over 65, there is a 30% risk of falling each year (57), and for those living in nursing homes, this rate increases to 50% (48). Falls in older adults can result in severe consequences, including fractures, immobilization, and even death (58). While regular physical activity reduces the risk of falls and related fractures (50), many older adults are unable to engage in such activities. NMES has, especially among older and/or untrained individuals, been shown to provide effective muscle activation, resulting in improved muscle strength and function (30, 31, 45, 54, 55, 59). For example, NMES treatment for 30 min, 2–3 times a week for 9 weeks has been shown to improve walking test time by 15%–20% (45).

#### Improved metabolic control

In addition to the risk of falls, physical inactivity also increases the risk of type 2 diabetes and obesity (50). Globally, one in eleven adults has diabetes, and in 2019 more than four million people died due to diabetes or its complications, equating to one death every 8 s. In addition to those already diagnosed with diabetes, even more people have pre-diabetes with the risk of developing the disease but also with a great opportunity for prevention (60). Physical activity is crucial both for prevention and treatment of type 2 diabetes, but as mentioned above, many are unable to engage in regular exercise. For these individuals, NMES presents as a valuable alternative, offering similar effects as regular physical activity on blood sugar regulation (53, 61–64). One study demonstrated that patients with type 2 diabetes who performed 40-min quadriceps NMES sessions, 5 days per week for 8 weeks, significantly improved fasting glucose levels and reduced body fat (53). A systematic review has confirmed these effects (65). Moreover, patients with type 2 diabetes often suffer from peripheral artery disease, which causes ischemic pain in the lower limbs and impairs walking. NMES has been shown to increase peripheral arterial flow, reduce ischemic pain, and enhance walking distances (66). However, systematic reviews call for more high-quality trials to draw definitive conclusions (67).

#### Preventing the formation of blood clots

Another significant risk posed by physical inactivity and extended immobilization is the development of blood clots in the legs or lungs (51). Between 1 and 4 out of 100 people will develop a blood clot requiring treatment during their lifetime (68). Anticoagulant treatments, while available, are not always effective (69), and for older adults who are prone to falls, they pose a bleeding risk (70). Mechanical compression therapy, such as intermittent pneumatic compression (IPC), is used in hospitals to increase blood flow, mimicking the muscle pump action that occurs during walking (71). However, IPC machines are too large and noisy for use outside of hospital environments, which is why NMES treatment, where electronics can be minimized, provides a quiet and mobile treatment option outside of hospitals. NMES treatment on the calf and quadriceps has been shown to improve venous blood flow in the leg vessels (12, 17, 18). Adding NMES treatment to drug therapy with anticoagulants during knee replacement surgery (72) and for patients undergoing major surgeries (73) reduces the risk of blood clots in the leg. While NMES alone can lower the risk of clots compared to no treatment during immobilization, it has not yet proven as effective as anticoagulants (18, 74). More research is needed to explore NMES as a sole treatment for preventing blood clots, as studies in this area are limited (18).

### Future directions

Future research should clarify the similarities and differences between the effects of NMES and regular physical exercise. One notable area of exploration is the load or impact on the cardiovascular system. Additionally, exercise has demonstrated neurobiological benefits, such as protection against cognitive disorders like dementia. A key factor released during exercise, which is brain protective, is brain-derived neurotrophic factor (BDNF). Interestingly, NMES has also been demonstrated to increased BDNF levels (75). The neurobiological effects of muscle stimulation are likely mediated via the release of myokines, which are peptide modulators of several tissue processes such as brain neuroplasticity, bone mineralization, and tissue repair (76). Notably, NMES has demonstrated the production of several myokines, which can exert beneficial effects on the pathophysiology of several conditions in patients with limited mobility (76). Future research should in more detail delineate the indications, settings and optimal dose-response relationships of NMES to induce beneficial effects.

### Limitations

While NMES shows promise, certain patients may be “non-responders” to NMES, particularly those with low contractile responses. Thus, in a study of critically ill patients in an intensive care unit it was demonstrated that patients with higher severity of illness were more likely to be non-responders to NMES (77). However, NMES has shown positive effects to maintain and improve limb strength in other severely ill populations, such as those with acute exacerbation of chronic obstructive pulmonary disease (78), acute heart failure (79), chronic kidney failure on hemodialysis (80) and spinal cord injury (81). Still, the existing studies have reported a wide range of stimulation parameters. Thus, future high-quality randomized trials should focus on standardizing NMES settings for specific indications.

## Conclusion

In summary, NMES offers a range of applications with positive effects for both older and younger individuals, including those who are healthy or living with health conditions. It can serve as a complement to physical activity or as an alternative when traditional exercise is not feasible, providing similar benefits. Additionally, NMES can also be considered as a non-invasive tool to address several research questions regarding muscles and muscle function in compromised populations. Although current NMES applications remain suboptimal, recent advancements in automated electrode placement and individualized stimulation settings show promise. These developments may improve treatment adherence and deepen our understanding of how to optimize NMES for various populations.
